# Assessing the Match Physical Responses of International Referees for Footballers with Cerebral Palsy: A Tournaments and Halves Comparative Analysis

**DOI:** 10.3390/s24051595

**Published:** 2024-02-29

**Authors:** Matías Henríquez, Eñaut Ozaeta, Daniel Castillo, Raúl Reina, María Isabel Cornejo, Aitor Iturricastillo, Skye Arthur-Banning, Javier Yanci

**Affiliations:** 1 Escuela de Kinesiología, Facultad de Odontología y Ciencias de la Rehabilitación, Universidad San Sebastián, Santiago 8380000, Chile; matias.henriquez@uss.cl; 2 Department of Physical Education and Sport, Faculty of Education and Sport, University of the Basque Country (UPV/EHU), 01006 Vitoria-Gasteiz, Spain; enaut.ozaeta@ehu.eus; 3 Valoración del Rendimiento Deportivo, Actividad Física y Salud, y Lesiones Deportivas (REDAFLED), Faculty of Education, University of Valladolid, 42004 Soria, Spain; daniel.castillo@uva.es; 4 Department of Sport Sciences, Sports Research Centre, Miguel Hernández University, 03202 Elche, Spain; 5 Escuela de Kinesiología, Universidad Santo Tomas, Santiago 8320000, Chile; isa.cornejo27@gmail.com; 6 Sports and Physical Exercise Research Group (GIKAFIT), Department of Physical Education and Sport, Faculty of Education and Sport, University of the Basque Country (UPV/EHU), 01006 Vitoria-Gasteiz, Spain; aitor.iturricastillo@ehu.eus (A.I.); javier.yanci@ehu.eus (J.Y.); 7 Department of Parks, Recreation, and Tourism Management, Clemson University, Clemson, SC 29634, USA; sarthur@clemson.edu

**Keywords:** soccer, team para-sport, referees, physical responses

## Abstract

Similar to conventional football, the modality dedicated to footballers with cerebral palsy (CP) requires referees who cope with the physical demands imposed during competitive matches to apply the rules of the game. While a significant body of research has explored the physical demands on referees in mainstream football, there is a noticeable lack of data regarding CP football. This study aimed to examine the physical response of international referees participating in different levels of world competitions for footballers with CP. Thirteen international referees, who officiated 49 matches in the men’s 2022 World Cup (1st to 15th ranked teams) and 2022 World Championships (16th to 30th ranked teams), participated in this study. A cross-sectional design was used to determine the physical responses and compare the 1st and 2nd halves and the performance in the different tournaments, recording physical variables throughout the matches. Significant higher physical responses were observed in the World Cup in comparison to the World Championship. Overall, high-level tournaments have been shown to elicit more intense physical responses from referees officiating CP football matches compared to lower-level tournaments. For the World Cup, a significantly higher number of accelerations and decelerations were registered in the 1st half compared to the 2nd half. This information may be useful for the strength and conditioning coaches of referees to plan weekly training sessions more specifically and adjust the periodical training load and post-match recovery protocols.

## 1. Introduction

Cerebral palsy (CP) is the most common health condition that produces motor disability in childhood and is one of the largest participant groups in para-sport [1]. Considering that football is one of the most popular sports worldwide, this discipline is widely practiced at a variety of competitive levels [2]. CP football is an expression of the regular sport, dedicated exclusively to players with CP and other related health conditions of brain injuries generating mild impairments of hypertonia, ataxia, or dyskinesia [3]. The professionalization of para-sport has increased the attraction of researchers studying the different factors that influence performance and improving the data to support the preparation of athletes with disabilities. Over the last decade, a significant number of studies have provided insight into the physical and physiological responses of football players with CP, offering a framework for performance and training [4,5,6,7]. Although most of the literature is focused on the performance of para-footballers, scarce information is available regarding referees, generating a gap in current knowledge when considering the personnel responsible for regulating the competition. 

Referees who officiate international level CP football matches supervise the application of minimal adaptation rules to allow the competition with modifications to include the field size, the number of players, no off-side law, small sized goals (i.e., 2 m × 5 m), permitting a throw-in with one hand, reduced total time, and a classification system that determines player participation according to the impact of the impairment in football skills [8]. Following the guidelines set by the International Federation of CP Football, officiating in this Para sport requires referees to attain the highest level of certification within their respective countries and to obtain a specific accreditation from the governing body (i.e., IFCPF) that requires specific training, certification, and reaccreditation. The certification must be renewed annually, and the referees must demonstrate successful completion of FIFA’s mandated physical tests, a track record of officiating matches at the top national level, and prior experience in officiating CP football matches. Although people with CP reported lower physical performance compared to the able-bodied population [9], the para-footballers running activity profile during competitions can reach considerable requirements of high-intensity actions [4,5,6,7], an aspect that referees should be physically prepared to face in this sport. Similar to regular football, CP football matches require a field referee and two assistant referees to supervise the game and ensure optimal positioning to facilitate decision-making, considering the specific impairment characteristics of the players and modifications of the para-sport [10]. Early studies in CP football reported a higher prevalence of goals scored per match compared to non-impaired competitions (e.g., 5 goals vs. 2 goals), which may be attributed to differences in field size, functional profiles, and contextual factors that can influence the final result of a match, and therefore the physical performance of the referee is crucial for the decisions on the game [8,11,12]. Referees of regular football move approximately 10 km during matches, of which almost 3 km are covered at a high intensity (over 13 km/h) necessary to follow the game situation, make adequate decisions, and respond to the physical demands of the match [10,13,14,15]. On the other hand, referees typically experience physiological responses that involve maintaining intensities approximately between 70–90% of their maximum heart rate, requiring high aerobic demands to officiate competitive matches [12]. However, to date, little research has reported the physical responses of referees officiating CP football matches.

Currently, CP football presents a structured program of competition including world and regional tournaments divided by the level of performance, providing for ever increasing professionalization of the environments in accordance with the rising participation of para-footballers. Due to the implications of the referee decisions in the outcome of the game and the relevant role played in football [12], a deeper understanding of the running capacities of referees of CP football may support the characterization of the responses encountered during competitive matches and support referee mentors and coaches to implement optimal training programs. Therefore, the first aim of this study was to describe and compare the physical responses between World Cup and World Championships tournaments in referees of CP football. Given that tournaments enrol teams with different ranking levels, it is hypothesized that the physical responses of referees recorded in the World Cup and World Championships may differ. The second aim of the study is to compare the referees’ physical responses between the 1st and 2nd halves, hypothesising a decrease in the physical responses in the second halves, just as it occurs with referees in 11-a-side mainstream football.

## 2. Materials and Methods

### 2.1. Participants

Thirteen international referees (39.17 ± 8.26 years; 76.07 ± 13.39 kg; 174.04 ± 9.97 cm; 24.99 ± 3.28 kg/m^2^) from three continents and eleven different nationalities who officiated the men’s 2022 World Cup (participation of teams between world ranking 1st to 15th held in Salou, Spain) and 2022 World Championships (participation of teams between world ranking 16th to 30th held in Olbia, Italy), voluntarily participated in this study. Of the total participants, 10 were men (40.22 ± 8.20 years; 80.32 ± 10.07 kg; 176.70 ± 6.45 cm; 25.74 ± 3.22 kg/m^2^; World Cup = 6; World Championships = 5) and 3 were women (36.00 ± 5.89 years; 61.90 ± 10.84 kg; 165.17 ± 12.52 cm; 22.50 ± 0.76 kg/m^2^; World Cup = 2; World Championships = 2), with 18.62 ± 9.24 and 6.54 ± 4.22 years of experience refereeing regular football and CP football, respectively. Of the total participants, 8 officiated World Cup matches (37.13 ± 6.53 years; 80.46 ± 13.09 kg; 175.50 ± 10.73 cm; 26.06 ± 3.59 kg/m^2^) and 7 officiated World Championships matches (43.83 ± 8.64 years; 73.33 ± 12.70 kg; 173.36 ± 8.48 cm; 24.30 ± 3.13 kg/m^2^). Two referees were able to participate in both tournaments. None of the evaluated referees presented with any physical or physiological limitations to conduct their officiating duties. In addition, all were informed about the study protocols and gave their informed consent to take part in this study. This investigation was approved by the Ethics Committee of the principal investigator’s university (reference no. DPS.RRV.03.17).

### 2.2. Procedures

A cross-sectional design was used to determine the physical responses to compare the 1st and 2nd halves and analyse the performance between the different tournaments, recording physical variables throughout the matches. Data were collected for 15 independent referee observations extracted from 49 valid matches played between 09:00 to 18:00 h in the world competitions. For the comparison between tournaments, 31 observations (total time = 68.76 ± 8.41 min) in 8 international referees and 18 observations (total time = 66.85 ± 8.50 min) in 7 international referees were included for the World Cup and World Championships, respectively. All of the referees used vests designed to wear a global positioning system (GPS) device to monitor the physical responses. In preparation for the matches, participants avoided any type of extenuating exercise and performed a standardized 15-min warm-up. The matches were played in two different venues on artificial turf with a field size of 50 m × 70 m.

### 2.3. Measures

Match running performance was recorded using GPS devices (WIMU PROTM, RealTrack System SL, Almería, Spain), sampling at a frequency of 10 Hz. A previous study reported the validity and reliability of this device to assess physical performance [16], which was used in able-bodied football players [17], football players with CP [18], and referees of regular football [19]. Before the official matches, the devices were activated and synchronized according to the manufacturer’s recommendations. All of the collected data were extracted using S PRO+^TM^ v. 2.2.2 software (RealTrack Systems, Almeria, Spain). The participants wore a GPS unit inserted in a purpose-built harness secured between the upper scapula with a fitted body vest. The variables considered for the analysis were described in each match half and match total time: total distance (TD) covered, explosive distance (ED), total distance covered with acceleration above 1.12 m/s^2^ in m) [19], maximum speed (km/h), distance covered at different speeds (walking: 0–6 km/h; jogging: 6–12 km/h; medium intensity running [MIR]: 12–18 km/h; high intensity running [HIR]: 18–21 km/h; sprinting: 21–24 km/h; and maximum sprinting [MS]: >24 km/h) [18], high-intensity breaking distance (HIBD: distance decelerating >2 m/s^2^), total number of sprints (n), sprinting distance (m), high-speed running distance (HSRD; >15.1 km/h in m), high-speed running actions (HSRA: n) [20]. Moreover, the total number of accelerations and decelerations (where an acceleration or deceleration is deemed to be any increase or reduction in speed that means passing or descending from the zero axis), maximal acceleration (m/s^2^), and maximal deceleration (m/s^2^) were registered [19]. In addition, the distance covered accelerating by zones (Z1: 0–1 m/s^2^; Z2: 1–2 m/s^2^; Z3: 2–3 m/s^2^; Z4: >3 m/s^2^), and distance covered decelerating by zones (Z1: 0–1 m/s^2^; Z2: 1–2 m/s^2^; Z3: 2–3 m/s^2^; Z4: >3 m/s^2^) were considered for the analysis [21]. Finally, the player load was used and represented in arbitrary units [17].

### 2.4. Statistical Analysis

Results are presented as mean ± standard deviation. Normal distribution and the homogeneity of variances were tested using the Kolmogorov-Smirnov and Levene tests. Student’s t-test for independent samples was performed to evaluate mean referees’ physical response differences between the World Cup and World Championships. Student’s t-test for paired samples was performed to evaluate mean differences between the 1st and 2nd half of CP football referees’ physical responses. Practical significance was assessed by Cohen’s d [22], with values of above 0.8, between 0.8 and 0.5, between 0.5 and 0.2, and lower than 0.2 being considered large, moderate, small, and trivial, respectively. The 95% confidence intervals of the effect sizes were calculated with their respective upper and lower boundaries. Data analyses were performed using JASP (JASP for Windows, version 0.13, Amsterdam, The Netherlands) and the Statistical Package for Social Sciences for Windows (version 24.0; SPSS Inc., Chicago, IL, USA). Statistical significance was set at *p* < 0.05 to reject the null hypothesis.

## 3. Results

**Figure 1:**
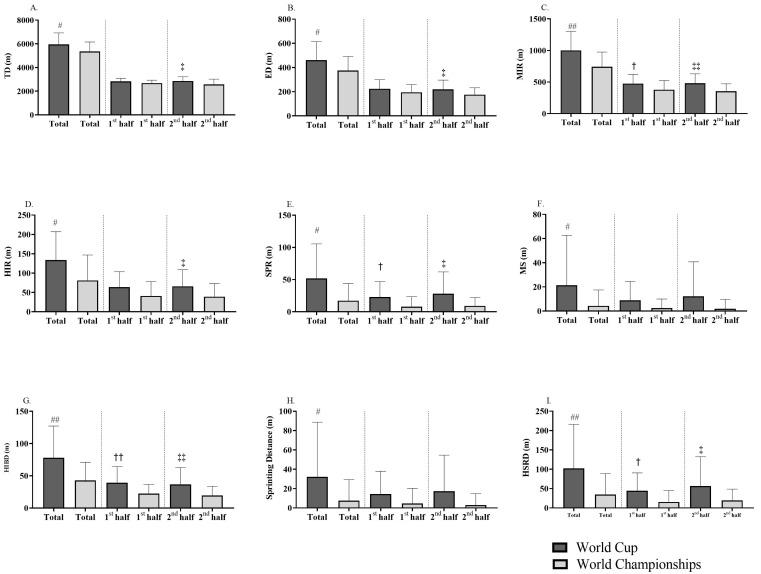
Comparison of the physical response variables by referees in cerebral palsy football matches related to the total distance covered (**A**), explosive distance (**B**), medium intensity running (**C**), high intensity running (**D**), sprinting (**E**), maximum sprinting (**F**), high-intensity breaking distance (**G**), sprinting distance (**H**) and high-speed running distance (**I**) in both World Cup and World Championships halves. TD: total distance; ED: explosive distance; MIR: medium intensity running; HIR: High intensity running; SPR: sprinting; MS: maximum sprinting; HIBD: high-intensity breaking distance; HSRD: high-speed running distance. # Significant differences in total time between World Cup and World Championships (## *p* < 0.01; # *p* < 0.05); † Significant differences in 1st half between World Cup and World Championships (†† *p* < 0.01; † *p* < 0.05); ‡ Significant differences in 2nd half between World Cup and World Championships (‡‡ *p* < 0.01; ‡ *p* < 0.05).

Regarding the comparison of the physical response of field referees during the different competitions (Figure 1), the TD and ED values were higher in the World Cup in both the total time (*p* < 0.05; d = 0.62 to 0.65; moderate) and 2nd half (*p* < 0.05; d = 0.62 to 0.72; moderate) and maximum speed values were higher in total time (*p* = 0.01; d = 0.80; large), 1st half (*p* < 0.01; ES = 0.90; large), and 2nd half (*p* = 0.03; d = 0.67; moderate). In addition, referees performed a higher distance in the total time, 1st half, and 2nd half at MIR (*p* < 0.05; d = 0.67 to 0.93; moderate to large), sprinting (*p* < 0.05; d = 0.67 to 0.76; moderate), HIBD (*p* < 0.01; d = 0.77 to 0.81; moderate to large), HSRD (*p* < 0.05; d = 0.59 to 0.71; moderate), and number of HSRA (*p* < 0.05; d = 0.55 to 0.67; moderate) in World Cup matches compared to World Championships matches. Similarly, a greater distance was covered in World Cup matches as compared to the World Championships matches at HIR in total time and 2nd half (*p* < 0.05; d = 0.68 to 0.74; moderate), MS in total time (*p* = 0.04; d = 0.50; moderate), number of sprints in total time and 2nd half (*p* < 0.05; d = 0.52; moderate), and sprinting distance in total time (*p* = 0.04; d = 0.52; moderate). No significant differences were found for player load (*p* > 0.05).

Table 1 shows the total distance covered, maximum speed, and distance covered at different intensities, as well as the differences between both halves of the World Cup and World Championships. The results showed no significant differences in physical responses between the 1st and 2nd halves of the played matches (*p* > 0.05; d = 0.06 to −0.28; trivial to small) when considering all of the tournaments and only significant differences between the 1st and 2nd half were found in the distance covered jogging in the World Championships (*p* = 0.03; d = 0.55; moderate), being higher in the 1st halves. No significant differences were observed between 1st and 2nd halves in World Cup matches.

**Figure 2:**
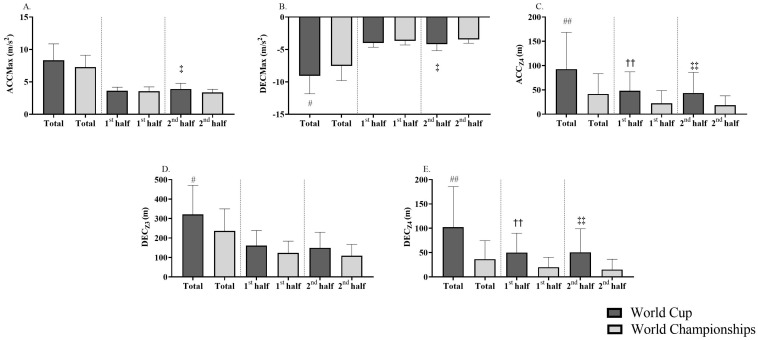
Physical response variables by referees in cerebral palsy football matches related to maximal acceleration (**A**), maximal deceleration (**B**), distance covered at very high acceleration (**C**), distance covered at high deceleration (**D**), and distance covered at very high deceleration (**E**) in both halves of the World Cup and World Championships. ACC_MAX_: maximum acceleration; DEC_MAX_: maximum deceleration; Z1: low acceleration/deceleration (0–1 m/s^2^); Z3: high acceleration/deceleration (2–3 m/s^2^); Z4: very high acceleration/deceleration (>3 m/s^2^). # Significant differences in total time between World Cup and World Championships (## *p* < 0.01; # *p* < 0.05); †† Significant differences in 1st half between World Cup and World Championships (†† *p* < 0.01); ‡ Significant differences in 2nd half between World Cup and World Championships (‡‡ *p* < 0.01; ‡ *p* < 0.05).

With respect to the comparison between the two tournaments and the short-term actions, a similar pattern was presented in which referees during the World Cup performed more maximal accelerations in the 2nd half (*p* = 0.03; d = 0.68; moderate), and more maximal decelerations in the total time and 2nd half (*p* < 0.05; d = −0.60 to −0.85; moderate to large) (Figure 2). Additionally, CP football referees covered more distance accelerating (*p* < 0.05; d = 0.70 to 0.78; moderate) and decelerating in Z4 (*p* < 0.05; d = 0.87 to 0.94; large) in both halves and total time during World Cup matches compared to World Championships. However, deceleration in Z3 only was significantly higher in the total time (*p* < 0.05; d = 0.62; moderate).

Concerning the analysis between halves, referees in the World Cup tournament showed a higher number of accelerations (*p* = 0.01; d = −0.55; moderate) and decelerations (*p* < 0.01; d = −0.54; moderate) in the 2nd half compared to the 1st half (Table 2). In addition, the distance accelerating at Z1 (*p* = 0.04; d = −0.38; small) was significantly greater in the 2nd half compared to the 1st half. For the World Championships the maximal decelerations (*p* = 0.04; d = −0.52; moderate) and deceleration in Z2 (*p* = 0.03; d = −0.58; moderate) were significantly greater in the 1st half compared to the 2nd half.

## 4. Discussion

The purpose of this study was to examine the physical responses of field international referees of CP football during different matches according to the level of competition (World Cup or World Championship) and considering total time. Previous studies have sought to understand referees’ physical responses to non-impaired football based on elements of the level of competition [13], their position on the field, as a referee or an assistant referee [13,15,23], contextual factors that may have been at play [24], and a host of other situations that may have taken place in each of the halves of a game. To the best of the authors’ knowledge, this is the first study to analyse the differences in physical responses between tournaments and halves in referees of CP football.

The results of this study showed that CP football referees covered a distance of 5731.82 ± 950.00 m of which 2591.58 ± 288.54 m was walking, 2066.24 ± 518.78 m was jogging, 905.62 ± 303.00 m was at medium intensity, 114.22 ± 74.64 m was at high intensity, 39.04 ± 48.28 m was sprinting, and 15.13 ± 34.61 m was at maximum sprinting. Previous studies on regular football referees officiating international matches showed that referees covered on average between 10.300 and 11.300 m [15,25]; however, since regular football matches last 90 min, referees have more time to cover that distance. However, converting to the 60 min duration of a CP football match, referees would cover an average distance between 6.901 and 7.571 m, a comparably higher distance. Nevertheless, the distances covered by amateur referees in regular football are comparably less, averaging between 8.650 and 9.990 m per match [26,27]. When converting to the 60 min duration of a CP football match, amateur referees of regular football cover a distance between 5.795 and 6.693 m, a greater comparable distance than that covered by CP football referees, although closer to the values registered by them. Notwithstanding, previous studies carried out with referees are in 11-a-side football, whose minimum dimensions are 45 × 90 m, while CP football is played on 7-a-side fields, whose dimensions are 50 × 70 m and the size of the field of play has already been shown to influence amateur refereeing, with the smallest fields of play being the ones where referees have registered the least physical responses [24]. This may be one of the reasons for the differences found in the physical responses recorded by referees in football and CP football matches.

Competitive sports such as CP football require a considerable number of acceleration and deceleration actions, generating higher mechanical and metabolic loads necessary to fulfil technical and tactical needs [28]. In the present study, referees of CP football performed 1839.04 ± 261.66 accelerations and 1848.64 ± 277.48 decelerations per match. In contrast, previous studies in regular football have shown a higher number of accelerations and decelerations per game ~2813.02 and ~2813.16 [29], although converting to the 60 min duration of a CP football match, referees would perform an average of 1884 accelerations and decelerations per game. Taking into account that the duration of a regular football match is 90 min as compared to 60 min for the adapted modality, it is reasonable to suggest that the accelerations per minute performed by referees in CP football are similar to the accelerations performed by regular referees. International games place demands on acceleration and deceleration actions highlighting that referees must be physically prepared to keep up with the game at any level of international play [12]. Interestingly, the obtained results in maximum speed (22.04 ± 2.69 km/h) were similar to those described by regular football referees, registering ~20.86 km/h [30], ~25.47 km/h [31], and ~19.40 km/h [32]. However, lower short-term actions were presented in CP football referees as compared to the performance of referees in regular football which might be due to the specific characteristics, the field size, and the demands of the para-sport. Furthermore, player load has been indicated as a variable that involves the sum of the accelerations in different planes and is commonly used to understand demands in football. In this regard, no significant differences were found between tournaments and between halves, which may lead us to believe that the use of this metric could be useful with an individualized approach to understanding the referees’ load [17].

Significant differences were present in physical response variables when the different tournaments were analysed, demonstrating a pattern in which the match’s physical demands on referees were higher in the tournament that involved teams of a higher ranking (i.e., World Cup). Previous studies on regular football referees have shown that in the higher-level categories, the physical responses of referees were similarly higher [29,33]. It is likely that contextual factors such as the level of competition can be a variable that influences the physical response, however with the particular analysis used, it is not possible to infer this causation. In regular football, it has been shown that the physical response of referees is largely based on the players’ performance metrics [34,35]. In this regard, Henríquez et al. [11], explored the difference in the physical response of international footballers with CP, suggesting that players from top-ranked teams playing against teams of similar level reported higher physical demands, as did bottom-ranked teams playing against teams of equivalent rank. In much that same way, the physical responses of the referees who officiated World Cup matches have been higher than the physical responses registered by the referees of the World Championships, given that in conventional football, it has also been observed that the players of higher competitive level register higher physical responses [36,37]. The fact that players belong to a higher competitive level implies that they are exposed to greater physical responses [36,37]. The present results suggest that referees must, at minimum, be prepared for the base level requirements demanded by the type of competition but ultimately should be prepared for the highest levels of demands that the competition can provide in order to meet the demand of the players they are officiating. The level of the tournament, despite being top-level, could influence the physical responses of the referees. Refereeing higher-level teams may require greater physical performance, something that should be considered in CP football tournaments, where referees officiate for several consecutive days.

When analysing differences between game halves, no differences were found in the total values of the variables in the present study. However, for the World Cup referees performance, significant differences were found in accelerations, decelerations, and Z1 (accelerations), with these being higher in the 2nd half. On the other hand, in the World Championships, referees covered a greater distance jogging in the 1st half. Additionally, differences were found in maximum deceleration variables and Z2 (decelerations), with these being higher in the first half of the match. While the findings have been mixed in the existing literature with respect to these variables, there have been some studies that demonstrated that physical responses were reduced in the 2nd half [25,38], as was the case for the World Championship in this study. More recent studies have shown that there were no differences in the distance covered between the 1st and 2nd halves [13,29,30,39], which is consistent with what is reported in the total values of the observations in this study. The negative influence of eligible impairments in footballers with CP have been extensively studied showing diminished physical performance as compared to footballers without CP [2,9,40,41]. It would make sense then that the demands on the referees would mirror the demands of the game and the athletes within the game for both 11v11 and 7v7 CP football games. While this analysis was not done in this study, additional research could be undertaken to explore the distinctions between the first and second halves within the same tournament and a comparison between the demands on the CP Football referee and the regular 11v11 referee as compared to the demands on the players in each of the two distinct versions of the game. This study is not without limitations. First, it utilizes absolute values to assess the physical responses of the referees during matches without accounting for individual fitness levels, potentially biasing the interpretation of results. Moreover, some referees officiated more than one analysed match in this study, so this and other personal factors could potentially influence the findings of the present study. Second, the reported skill and physical level of players in both tournaments could potentially impact the results obtained. And third, physiological responses related to physical responses were not considered in this study (e.g., heart rate, blood pressure or oxygen consumption). Nevertheless, future studies should address these limitations by considering them and exploring the physical and/or physiological responses of referees in CP football in detail and considering the possible interaction between tournament level and referees’ performance.

## 5. Conclusions

This study has described the physical responses that referees of CP football endure during international tournaments, thus providing new information for physical trainers to adjust weekly-monthly training loads. The results of the present study show that international top-level tournaments require greater physical responses from referees who officiated CP football matches than lower-level tournaments, in terms of covering more total distance, more explosive distance, and greater distances covered at various intensities during the first and second halves. Therefore, the results suggest that the physical responses of officiating top-level CP football matches are higher than officiating lower-level matches. Similarly, there were small differences noted between the two halves of the match in that there are more accelerations and decelerations in the second halves of matches, although only a moderate effect size was noted.

## Figures and Tables

**Table 1:** Physical response variables by referees in cerebral palsy football matches related to the total distance covered, maximum speed, and distance covered at different intensities in both World Cup and World Championships halves.

	Total Sample (*n* = 13 Referees, 49 Observations)				World Cup (*n* = 8 Referees, 31 Observations)				World Championships (*n* = 7 Referees, 18 Observations)			
Variables	Total	1st Half	2nd Half	*d* (95% CI)	Total	1st Half	2nd Half	*d* (95% CI)	Total	1st Half	2nd Half	*d* (95% CI)
TD (m)	5731.82 ± 950.00	2774.90 ± 264.90	2753.66 ± 413.67	0.07 (−0.21, 0.35)	5950.98 ± 970.83	2828.40 ± 264.14	2857.33 ± 362.73	−0.12 (−0.47, 0.24)	5354.37 ± 804.07	2682.77 ± 246.57	2575.11 ± 444.55	0.27 (−0.21, 0.73)
ED (m)	430.11 ± 144.29	213.79 ± 71.83	203.30 ± 72.98	0.24 (−0.05, 0.52)	461.85 ± 151.28	224.76 ± 74.60	219.38 ± 77.06	0.13 (−0.23,0.48)	375.43 ± 115.73	194.89 ± 64.44	175.61 ± 57.21	0.41 (−0.08, 0.89)
Max speed (km·h)	22.04 ± 2.69	22.96 ± 2.91	22.10 ± 2.83	0.08 (−0.21, 0.36)	22.79 ± 2.61	23.14 ± 2.75	22.78 ± 2.82	0.16 (−0.19, 0.52)	20.76 ± 2.38	20.73 ± 2.58	20.95 ± 2.52	−0.14 (−0.60, 0.33)
Distance at different intensities												
W (m)	2591.58 ± 288.54	1242.56 ± 122.30	1260.17 ± 171.94	−0.14 (−0.42, 0.15)	2606.57 ± 310.76	1238.86 ± 106.34	1261.29 ± 129.06	−0.24 (−0.59, 0.12)	2565.75 ± 504.69	1248.92 ± 149.02	1258.25 ± 232.53	−0.05 (−0.51, 0.41)
J (m)	2066.24 ± 518.78	1013.38 ± 179.34	974.32 ± 231.76	0.26 (−0.03, 0.54)	2136.85 ± 570.34	1018.44 ± 192.78	1009.25 ± 219.04	0.07 (−0.29, 0.42)	1944.63 ± 401.40	1004.67 ± 158.42	914.16 ± 246.84	0.55 (0.04, 1.04) *
MIR (m)	905.62 ± 303.00	439.74 ± 151.73	433.43 ± 150.58	0.05 (−0.23, 0.33)	1000.96 ± 300.90	475.64 ± 146.61	480.42 ± 148.62	−0.04 (−0.39, 0.31)	741.42 ± 233.08	377.91 ± 143.80	352.51 ± 118.65	0.20 (−0.27, 0.66)
HIR (m)	114.22 ± 74.64	55.28 ± 40.31	56.12 ± 41.59	−0.02 (−0.30, 0.26)	133.48 ± 73.64	63.66 ± 40.19	66.01 ± 42.92	−0.06 (−0.41, 0.29)	81.06 ± 65.72	40.86 ± 37.25	39.08 ± 33.88	0.06 (−040, 0.53)
SPR (m)	39.04 ± 48.28	17.37 ± 22.19	21.20 ± 29.29	−0.20 (−0.48, 0.09)	51.73 ± 53.62	22.85 ± 23.85	28.15 ± 33.76	−0.23 (−0.59, 0.13)	17.18 ± 26.63	7.94 ± 15.44	9.24 ± 13.10	−0.12 (−0.59, 0.34)
MS (m)	15.13 ± 34.61	6.57 ± 13.54	8.42 ± 23.57	−0.11 (−0.39, 0.17)	21.39 ± 41.33	8.95 ± 15.65	12.22 ± 28.50	−0.16 (−0.52, 0.19)	4.33 ± 13.12	2.47 ± 7.55	1.87 ± 7.94	0.07 (−0.39, 0.53)
HIBD (m)	65.02 ± 45.65	32.98 ± 22.93	30.40 ± 23.85	0.22 (−0.06, 0.51)	77.83 ± 49.27	39.16 ± 24.92	36.77 ± 26.08	0.18 (−0.18, 0.54)	42.96 ± 28.02	22.34 ± 14.13	19.43 ± 14.28	0.36 (−0.12, 0.83)
Sprints (n)	1.33 ± 2.54	0.67 ± 1.33	0.63 ± 1.44	0.04 (−0.24, 0.32)	1.84 ± 2.95	0.90 ± 1.47	0.90 ± 1.68	0.00 (−0.35, 0.35)	0.44 ± 1.29	0.28 ± 0.96	0.17 ± 0.71	0.10 (−0.36, 0.57)
Sprinting distance (m)	23.04 ± 48.12	10.81 ± 21.31	11.98 ± 31.16	−0.05 (−0.33, 0.23)	32.06 ± 56.61	14.37 ± 23.52	17.30 ± 37.32	−0.11 (−0.46, 0.24)	7.51 ± 21.89	4.68 ± 15.56	2.83 ± 12.00	0.11 (−0.36, 0.57)
HSRA (n)	4.41 ± 5.13	1.94 ± 2.32	2.41 ± 3.17	−0.21 (0.50, 0.07)	5.61 ± 5.64	2.48 ± 2.47	3.03 ± 3.60	−0.22 (−0.57, 0.14)	2.33 ± 3.31	1.00 ± 1.71	1.33 ± 1.91	−0.22 (−0.69, 0.25)
HSRD (m)	77.47 ± 100.91	33.84 ± 42.78	42.66 ± 65.21	−0.20 (0.48, 0.09)	102.33± 113.62	44.52 ± 45.95	56.29 ± 76.10	−0.22 (−0.57, 0.14)	34.63 ± 53.82	15.45 ± 29.59	19.18 ± 29.41	−0.16 (−0.62, 0.31)

Note. TD: total distance; ED: explosive distance; W: walking distance (0–6 km/h); J: jogging distance (6–12 km/h); MIR: medium intensity running (12–18 km/h); HIR: high intensity running (18–21 km/h); SPR: sprinting (21–24 km/h); MS: maximum sprinting (>24 km/h); HIBD: high intensity braking distance; HSRA: high-speed running actions. * Significant differences between 1st half and 2nd half (*p* < 0.05).

**Table 2:** Physical response variables by referees in cerebral palsy football match related to the number of accelerations, deceleration, maximal acceleration, maximal deceleration, distance covered accelerating and decelerating at different speeds, and player load in both halves of the World Cup and World Championships.

	Total Sample (*n* = 13 Referees, 49 Observations)				World Cup (*n* = 8 Referees, 31 Observations)				World Championships (*n* = 7 Referees, 18 Observations)			
Variables	Total	1st Half	2nd Half	*d* (95% CI)	Total	1st Half	2nd Half	*d* (95% CI)	Total	1st Half	2nd Half	*d* (95% CI)
Accelerations (n)	1839.04 ± 281.66	870.18 ± 94.60	904.90 ± 113.65	−0.28 (−0.57, 0.01)	1839.13 ± 265.64	852.81 ± 70.38	907.68 ± 93.41	−0.55 (−0.93, −0.17) **	1838.89 ± 315.39	900.11 ± 122.59	900.11 ± 145.04	0.00 (−0.46, 0.46)
Decelerations (n)	1848.64 ± 277.48	875.16 ± 94.32	909.22 ± 112.90	−0.27 (−0.56, 0.01)	1848.23 ± 261.93	856.65 ± 69.09	912.32 ± 94.15	−0.54 (−0.91, −0.16) **	1849.44 ± 310.38	907.06 ± 122.44	903.89 ± 142.47	0.02 (−0.44, 0.48)
ACC_Max_ (m/s^2^)	7.94 ± 2.34	3.61 ± 0.59	3.71 ± 0.79	−0.13 (−0.41, 0.15)	8.33 ± 2.53	3.64 ± 0.54	3.90 ± 0.88	−0.30 (−0.66, 0.06)	7.28 ± 1.84	3.56 ± 0.67	3.38 ± 0.47	0.38 (−0.10, 0.86)
DEC_Max_ (m/s^2^)	−8.51 ± 2.68	−3.86 ± 0.68	−3.91 ± 0.94	0.06 (−0.22, 0.34)	−9.08 ± 2.76	−3.98 ± 0.67	−4.18 ± 1.00	0.22 (−0.14, 0.58)	−7.53 ± 2.28	−3.66 ± 0.65	−3.43 ± 0.60	−0.52 (−1.00, −0.02) *
Distance covered at different acceleration intensities												
Z1 (m)	1361.33 ± 266.37	646.38 ± 91.81	664.77 ± 119.32	−0.22 (−0.50, 0.07)	1395.52 ± 256.10	650.85 ± 96.90	681.89 ± 104.17	−0.38 (−0.74, −0.01) *	1302.46 ± 280.68	638.66 ± 84.44	635.29 ± 139.97	0.04 (−0.42, 0.50)
Z2 (m)	1052.97 ± 268.45	509.46 ± 102.27	502.68 ± 120.62	0.08 (−0.20, 0.36)	1091.34 ± 292.37	513.49 ± 110.09	522.74 ± 116.07	−0.12 (−0.48, 0.23)	986.88 ± 212.87	502.51 ± 89.80	468.14 ± 123.74	0.35 (−0.14, 0.82)
Z3 (m)	392.07 ± 171.52	194.15 ± 96.83	187.20 ± 87.16	0.10 (−0.18, 0.38	422.37 ± 194.37	206.21 ± 109.96	202.24 ± 97.19	0.06 (−0.30, 0.41)	339.88 ± 108.81	173.38 ± 66.46	161.29 ± 60.48	0.17 (−0.30, 0.64)
Z4 (m)	73.99 ± 69.34	38.79 ± 36.67	34.44 ± 37.53	0.17 (−0.12, 0.45)	92.78 ± 75.61	48.19 ± 39.00	43.61 ± 42.55	0.15 (−0.20, 0.51)	41.63 ± 41.66	22.61 ± 25.95	18.64 ± 19.09	0.22 (−0.25, 0.68)
Distance covered at different deceleration intensities												
Z1 (m)	1468.99 ± 253.22	697.37 ± 73.94	718.26 ± 114.96	0.23 (−0.51, 0.06)	1470.22 ± 261.91	692.48 ± 74.97	710.01 ± 103.88	−0.21 (−0.56, 0.15)	1466.89 ± 244.93	705.79 ± 73.49	732.46 ± 133.91	−0.26 (−0.72, 0.22)
Z2 (m)	1025.72 ± 242.67	498.85 ± 93.21	488.73 ± 113.45	0.12 (−0.16, 0.40)	1052.06 ± 279.31	490.97 ± 105.32	507.75 ± 114.94	−0.26 (−0.62, 0.10)	980.34 ± 158.62	512.43 ± 68.18	455.97 ± 105.99	0.58 (0.07, 1.07) *
Z3 (m)	290.19 ± 142.344	146.98 ± 73.70	134.40 ± 75.00	0.24 (−0.05, 0.52)	321.27 ± 149.97	160.75 ± 77.89	149.50 ± 80.12	0.20 (−0.16, 0.56)	236.65 ± 112.79	123.26 ± 60.73	108.38 ± 58.50	0.30 (−0.18, 0.77)
Z4 (m)	78.19 ± 76.62	39.00 ± 36.82	37.49 ± 43.83	0.06 (−0.23, 0.34)	102.38 ± 83.25	50.07 ± 39.84	50.56 ± 48.31	−0.02 (−0.37, 0.34)	36.52 ± 37.98	19.94 ± 20.44	14.98 ± 21.41	0.26 (−0.22, 0.72)
PL (AU)	60.13 ± 18.09	29.27 ± 7.51	28.62 ± 7.62	0.16 (−0.12, 0.44)	59.86 ± 22.01	28.40 ± 8.62	28.36 ± 8.78	0.02 (−0.34, 0.37)	60.59 ± 8.25	30.76 ± 4.95	29.06 ± 5.26	0.31 (−0.17, 0.78)

Note. ACC_MAX_: maximum acceleration; DEC_MAX_: maximum deceleration; Z1: low acceleration/deceleration (0–1 m/s^2^); Z2: medium acceleration/deceleration (1–2 m/s^2^); Z3: high acceleration/deceleration (2–3 m/s^2^); Z4: very high acceleration/deceleration (>3 m/s^2^); PL: player load. Significant differences between 1st half and 2nd half at ** *p* < 0.01; * *p* < 0.05.

## Data Availability

Data are contained within the article.
